# Reusing dental implants?: an experimental study for detecting the success rates of re-osseointegration

**DOI:** 10.1186/s40729-018-0130-x

**Published:** 2018-06-19

**Authors:** Murat Ulu, Erdem Kılıç, Emrah Soylu, Mehmet Kürkçü, Alper Alkan

**Affiliations:** 1 Faculty of Dentistry, Oral and Maxillofacial Department, İzmir Katip Celebi University, İzmir, Turkey; 2 Faculty of Dentistry, Oral and Maxillofacial Department, Bezmialem University, İstanbul, Turkey; 3 Faculty of Dentistry, Oral and Maxillofacial Department, Erciyes University, Kayseri, Turkey; 4 Faculty of Dentistry, Oral and Maxillofacial Department, Cukurova University, Adana, Turkey

**Keywords:** Dental implant, Osseointegration, Peri-implantitis, Surface characteristics

## Abstract

**Background:**

The aim of this study was to histomorphometrically compare the implant-host integration between retrieved implants and new implants.

**Methods:**

Jaws in 10 male beagle dogs were divided into four groups, and 36 dental implants were inserted into the jaws. In groups 1 and 2, experimental peri-implantitis was induced within 2 months after implant insertion. In group 1, surface decontamination of implants was achieved using air-flow and citric acid. In group 2, implants were sterilized with autoclave after air-flow and citric acid surface decontamination. Subsequently, these implants were inserted in contralateral jaws of the same dogs and a 3-month period was allowed for osseointegration. In group 3, the implants were removed from human jaws due to peri-implantitis and were inserted into dog jaws following surface cleaning protocol and sterilization with autoclave and a 3-month period was allowed for osseointegration. Group 4 was set as the control group. After the osseointegration period, all the animals were sacrificed. The degree of osseointegration in all groups was evaluated by evaluating the ISQ values and by using histomorphometric measurements.

**Results:**

Histological findings showed that bone-implant contact (BIC) percentage (mean ± SD) was 83.39% ± 6.37 in group 1, 79.93% ± 11.83 in group 2, 75.45% ± 9.09 in group 3, and 80.53 ± 5.22 in group 4. Moreover, the resonance frequency analysis (RFA) and ISQ values were similar in all four groups both before and after the implantation.

**Conclusions:**

The results of this experimental study indicated that there is no significant difference between new dental implants and re-used dental implants with regards to osseointegration around the implant.

## Background

Branemark et al. conducted the first experimental trial with titanium dental implants and created a new vision by defining the term “osseointegration” in the 1960s [1]. Despite the advances in implant technology and protocols and the accumulating evidence in the literature, implant failure/loss may still occur due to several reasons [2]. On the other hand, although dental implant therapy is a successful treatment option for edentulous patients, it may lead to undesired complications after the insertion of the implant such as implant mobility, radiolucency around the implant, and inflammation of peri-implant tissues, or subjective complaints from the patients [3]. Peri-implantitis is a major complication of implant treatment characterized by inflammation of the soft tissues surrounding implants combined with loss of bone [4]. If this complication is not treated appropriately, implant retrieval may be necessary. On the other hand, the primary reasons for an unsuccessful implant treatment include anatomical complexity, inexperience of the surgeon, poor oral hygiene, and smoking [5].

The most undesired complication in implant therapy is peri-implantitis which leads to retrieval of a dental implant. Similar to gingivitis and periodontitis, the main etiologic factor for peri-implant mucositis and peri-implantitis is microbial dental plaque. Once an implant is inserted, bacterial colonization begins to occur on its surface [6]. The primary goal in nonsurgical treatment of peri-implant mucositis and peri-implantitis is to eliminate or reduce the bacteria levels in the peri-implant area and, ultimately, to re-establish a clinically healthy environment. However, with conventional treatment modalities, it is often difficult to eradicate microorganisms from threads and rough surfaces [7]. Instead, a number of techniques including laser treatment, air abrasion, citric acid application and conventional mechanical therapy have been used in peri-implantitis therapy. Nevertheless, despite the use of different techniques, complete elimination of pathogens around the implants may not always be possible. In particular, adequate decontamination may not be achieved due to the difficulty of attaining sufficient access to all the dental implant surfaces.

In the present study, a novel approach for peri-implantitis treatment is described, in which the infected implants are removed and the surface treatment is performed extra-orally due to the difficulty of implant surface decontamination inside the bone, and the implants are inserted into the bone for a second time after decontamination.

The aim of this study was to evaluate the implant-bone integration after the removal of an infected implant from the bone and to compare the success rate of this approach with that of new implants by using resonance frequency analysis and histomorphometry.

## Methods

### Research design

**Fig. 1:**
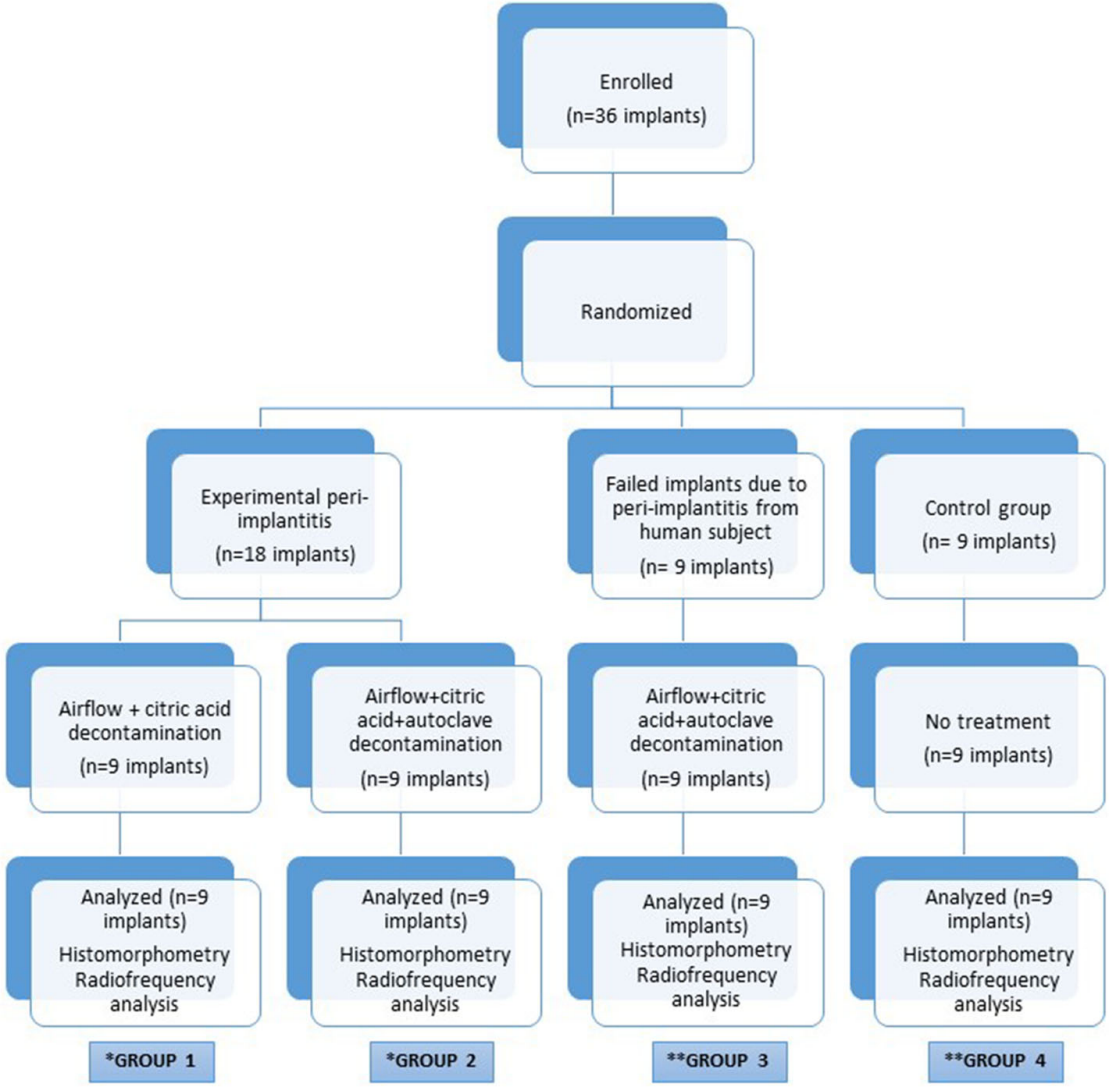
Flowchart of the research design employed in the study. *Three dogs were used in each group 1 and 2. Three implants were inserted right side of the mandibles. After peri-implantitis period, extracted implants were inserted into the left side of the mandibles. **Two dogs were used in each group 3 and 4. Six failed implants from human inserted into the one dog’s mandible bilaterally and three implants inserted into the other dog’s mandible unilaterally in group 3. Six implants inserted into the one dog’s mandible bilaterally and three implants inserted into the other dog’s mandible unilaterally in group 4 (control group)

This in vivo study had a comparative, randomized, prospective research design, and each group consisted of 10 male beagle dogs that were veterinarian-controlled, healthy, and of similar weight. Animal Research Reporting in Vivo Experiment (ARRIVE) guidelines were used, and surgical procedure was approved by the Local Animal Experiments Ethical Committee of Erciyes University. Adequate measures were taken to minimize the pain or discomfort in the animals. A total of 36 dental implants (tissue level, 3.3 × 10 mm, Straumann AG, Basel, Switzerland) were inserted in the animals according to the non-submerged healing protocol. Figure 1 presents the flowchart of the research design employed in the study.

### Sedation, anesthesia, animal care, and sacrifice

All the interventions were performed under general anesthesia. Enteral nutrition was stopped 12 h before the surgical procedure. General anesthesia was achieved with 2 mg/kg xylazine hydrochloride (i.m.) (Rompun, Bayer, Istanbul, Turkey) and 5 mg/kg ketamine hydrochloride (i.m.) (Alfamyne, Egevet, Izmir, Turkey). After the surgery, a 3-day antibiotic therapy with Streptomycin 0.5 g/day (I.E. Ulagay, Istanbul, Turkey) was administered in each dog. Postoperative care included daily observations regarding appetite and the documentation of adverse events such as bleeding, pain, swelling, and discomfort. At the end of the experiment, all the animals were sacrificed with a large dose of pentobarbital (i.v.). The animals in groups 3 and 4 were sacrificed at month 6 and the animals in groups 1 and 2 were sacrificed at month 8 after the extraction surgery.

### Surgical procedure

**Fig. 2:**
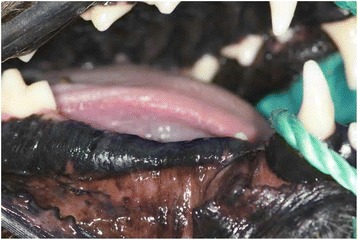
Edentulous posterior mandible of the dog at 3 months after tooth extraction

The surgical procedure was commenced by the extraction of the mandibular second, third, fourth pre-molars, and the first molar bilaterally. The pupillary reflex was controlled after the administration of anesthetic drugs. Peri- and intra-oral tissues were disinfected with 10% povidone-iodine solution, and the surgical area was covered with sterile covering. Infiltration anesthesia with 2% articaine (Ultracaine DS, Sanofi Aventis Drugs, Istanbul, Turkey) was applied to the premolar area for hemostasis and for post-operative pain control. A full-thickness vestibular flap was elevated gently, and surgical tooth extraction was performed using surgical burs with straight elevators. Surgical wounds were closed with 3/0 vicryl sutures and streptomycin 0.5 g/day was administered for 3 days postoperatively. After the extraction, a 3-month period was allowed for healing of the alveolar bone and soft tissue (Fig. 2).

After the 3 month healing period, a second surgery was performed for implant insertion. Pre-surgical procedures were the same as those described above. In addition, a horizontal incision was made along the edentulous premolar area. A mucoperiosteal flap was gently elevated to expose the recipient bone and the implant sockets were prepared using a commercially available surgical set (Straumann® instruments, Straumann AG, Waldenburg, Sweden) under sterile saline irrigation. All the implants had a sandblasted and acid-etched (SLA) surface and were of the same size and length (3.3 × 10 mm, tissue level). All implants were inserted to the level of the machined surface left below the bone. Non-submerged healing protocol was performed in all the groups and the flaps were closed with 3/0 vicryl sutures.

**Fig. 3:**
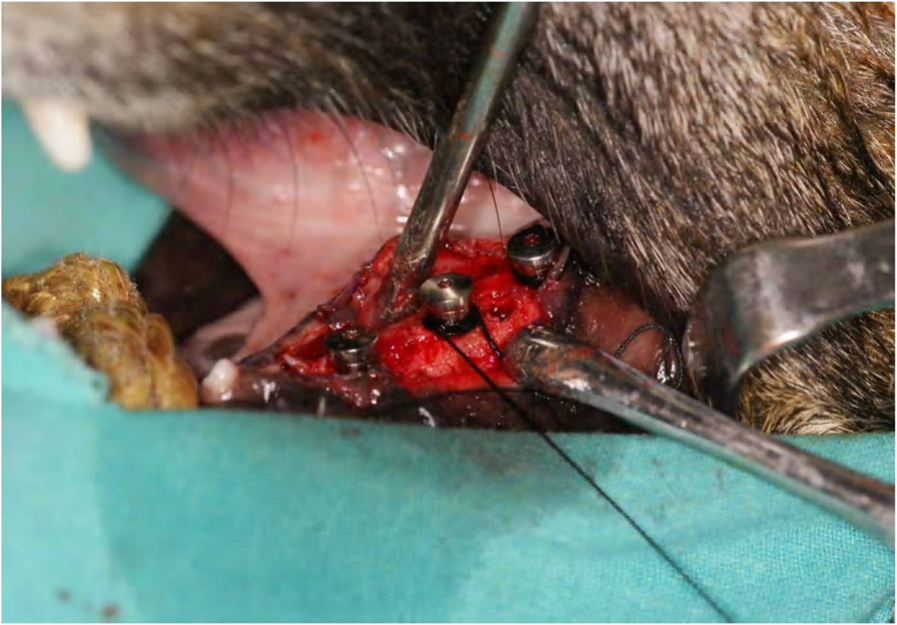
Silk ligatures placed in a submarginal position around the implants

**Fig. 4:**
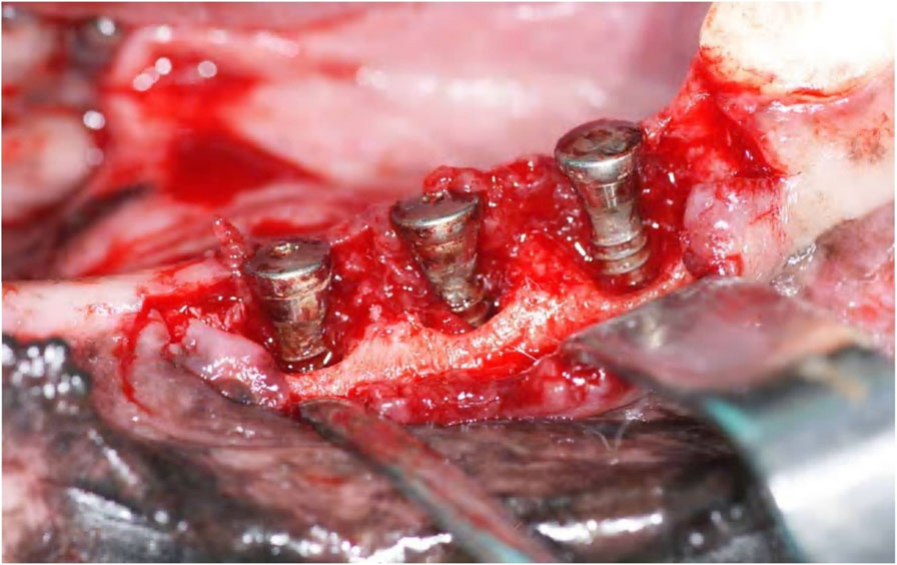
A 2-month period was allowed for plaque retention and peri-implantitis

In group 1, after the insertion of the implants, 3/0 silk sutures (Doğsan, Trabzon, Turkey) were placed below the free gingival/mucosal margin around the implants and plaque control was terminated for 2 months (Fig. 3). To promote plaque retention and peri-implantitis, the animals were fed a soft diet. The implants were removed with reverse torque after the induction of peri-implantitis (Fig. 4). After the removal, all the implant surfaces were cleaned by air-flow with bi-carbonate granules for 1 min prior to the treatment with citric acid (pH: 1). Subsequently, the implant surfaces were rinsed with sterile saline solution and then all the implants were inserted in the contralateral side of the mandible of the same dog. After a 3-month osseointegration period, the animals were sacrificed with a high dose of pentobarbital (i.v.).

In group 2, the same procedures were applied as in group 1. However, unlike the implants in group 1, the implants in group 2 were sterilized by autoclave treatment at 121 °C for 30 min. Afterwards, the sterilized implants were inserted in the contralateral side of the mandible of the same dog from which the implants were retrieved. After a 3-month osseointegration period, the animals were sacrificed with a high dose of pentobarbital (i.v.).

In group 3, failed implants due to peri-implantitis were obtained from human subjects. The surface of the implants were cleaned and sterilized with autoclave and then the implants were inserted into the mandibles of the dogs. After a 3-month osseointegration period, the animals were sacrificed with a large dose of pentobarbital (i.v.).

**Fig. 5:**
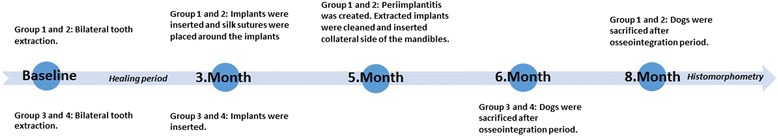
Time arrow about the stages of the study

In group 4 (control group), no implant insertion was performed and after a 3-month osseointegration period, the animals were sacrificed with a large dose of pentobarbital (i.v.). The preparation times, surgeries, and observation time points of all four groups were summarized on the time arrow (Fig. 5).

### Experimental design

#### Resonance frequency analysis (RFA) measurements

Implant stability was measured using resonance frequency analysis (RFA) with an Osstell® device (Osstell AB, Goteborg, Sweden). All implants were placed with non-submerged healing protocol and the Osstell® sensor was positioned perpendicular to the long axis of the implant in accordance with the guidelines provided by the manufacturer. The results were calculated in the form of objective ISQ values (ranging from 1 to 100). The RFA measurements were performed from four different directions (mesial, distal, lingual, and vestibule), and the mean ISQ was recorded as the final value.

#### Removal and preparation of the implant-bone specimens

The implants with a neighboring bone were removed en bloc, and the adhesive soft tissues were dissected to investigate the healing status and the bone-implant contact (BIC) percentage. The specimens were fixed in 10% neutral buffered formalin for 48 h and dehydrated in subsequent concentrations of 70–99.9% ethanol. After dehydration, the specimens were embedded in methyl methacrylate (Technovit 7200 VLC, Heraeus Kulzer GmbH & Co. KG, Wehrheim, Germany) without decalcification. 200-μm-thick slides were cut from the blocks using a band saw (Exakt 300 CL, Exakt Apparatebau, Norderstad, Germany).

#### Histologic and histomorphometric analysis

**Fig. 6:**
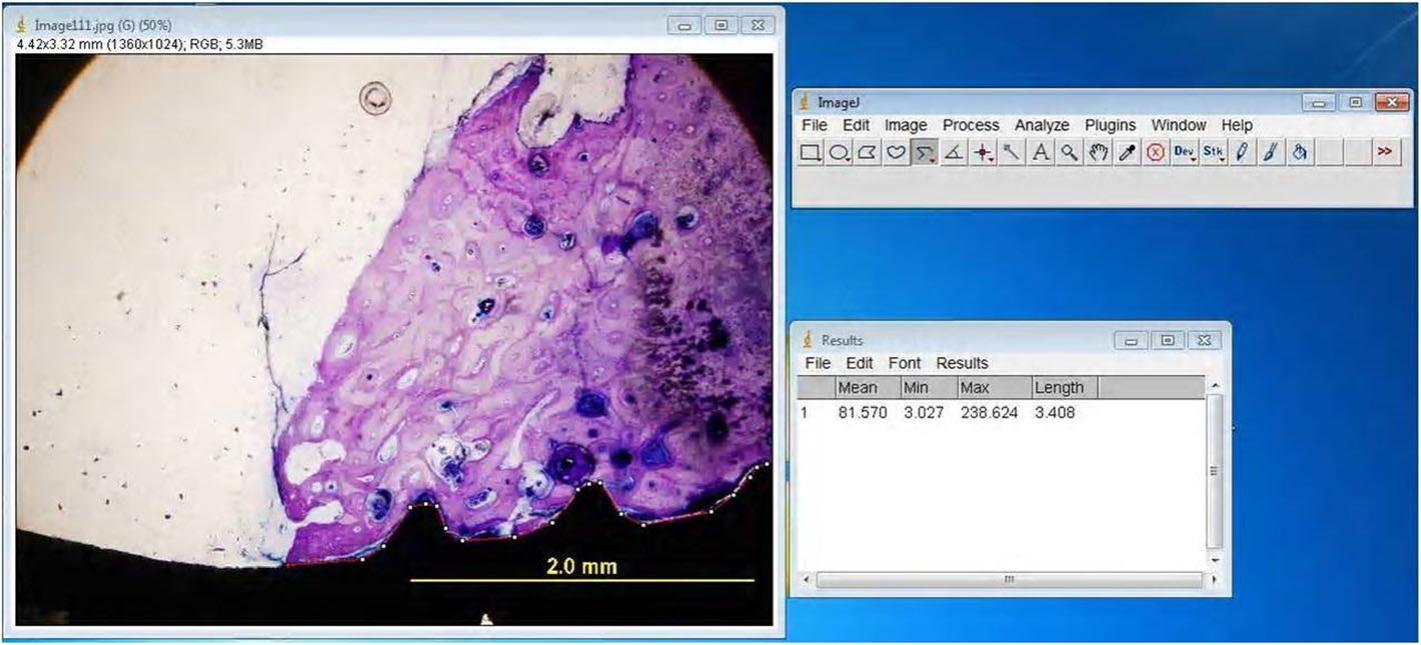
BIC percentage measured with ImageJ analysis software

The 50-μm-thick final histological slides were prepared by grinding with 320–4000 grit sandpapers (Hermes Schleifmittel GmbH & Co. KG, Hamburg, Germany). The final sections were mounted and stained with toluidine blue for histologic and histomorphometric analysis. In order to measure the BIC percentage, digital images of the sections were obtained by a digital camera (Olympus DP 70, Olympus, Tokyo, Japan) attached to a microscope (Olympus BX50). The obtained images were transferred to a computer and were histomorphometrically analyzed using ImageJ analysis software (ImageJ, National Institutes of Health, Bethesda, Maryland, USA) (Fig. 6). The BIC percentage was calculated by an experienced researcher blinded to the study protocol.

#### Statistical analysis

Statistical analyses were performed using SPSS v.20.0 (IBM, Chicago, IL, USA). The Shapiro-Wilks normality test was used to verify the normality of the data. All variables were normally distributed and thus parametric tests were used for intra-group (paired sample *t* test) and inter-group (one-way ANOVA/Tukey’s test) comparisons. A *p* value of < 0.05 was considered significant.

## Results

The experimental period and the laboratory workup of the study were unremarkable. Surgical operations were uneventful and the post-operative healing periods were completed with no complications. Histologic analysis and the ISQ values indicated that osseointegration was achieved in all the implants.

### Histomorphometric analysis

Histomorphometric analysis demonstrated that adequate bone formation in neighboring tissues was achieved in all four groups. In the histomorphometric analysis of the sections, the highest BIC percentage was seen in group 1 (83.39 ± 6.37) and the lowest BIC percentage was seen in group 3 (75.45 ± 9.09). However, no significant difference was found among the groups and all four groups were statistically similar with regards to BIC percentage. Tables 1 and 2 presents the histomorphometric measurements of the groups.

**Table 1:** Comparison of BIC percentages of over the entire implant length at 3-month follow-up

Group 1 [Mean ± SD]	Group 2 [Mean ± SD]	Group3 [Mean ± SD]	Group 4 [Mean± SD]	*p*
83.39 ± 6.37	79.93 ± 11.83	75.45 ± 9.09	80.53 ± 5.22	290^*^

*Statistically not significant

**Table 2:** Comparison of BIC percentages of 3 mm crestal area of the implants at 3-month follow-up

Group 1 [Mean ± SD]	Group 2 [Mean ± SD]	Group3 [Mean ± SD]	Group 4 [Mean ± SD]	*p*
77.67 ± 5.03	75.28 ± 10.65	71.86 ± 8.34	80.63 ± 5.58	.144^*^

*Statistically not significant

### RFA measurements

Table 3 presents the RFA measurements of the groups. In all four groups, the RFA measurements were performed after the insertion and before the removal of the implants. The highest RFA values after the insertion were observed in groups 3 (71.77 ± 5.71) and 4 (70.44 ± 5.15), whereas the highest RFA values before the insertion were observed in groups 2 (79.44 ± 2.55) and 4 (79.12 ± 4.61). No significant difference was established between the groups. Nevertheless, the only significant difference between the initial and final ISQ values was found in group 2.

**Table 3:** Inter- and intra-group ISQ analysis and measurements on day of surgery and at 3-month follow-up

	Mean ± SD ISQ day 0	Mean ± SD ISQ at 3 month	*p*
Group 1	69.33 ± 8.48	77.77 ± 1.78	.019
Group 2	68.88 ± 5.90	79.44 ± 2.55	.001^*^
Group 3	71.77 ± 5.71	75.11 ± 5.84	.366
Group 4	70.44 ± 5.15	79.12 ± 4.61	.022
*p*	.782	.115	

*Statistically significant

## Discussion

Approximately two million new dental implants are inserted per year around the world and tens of millions of implants are still in use. Moreover, it is estimated that approximately 200,000–250,000 implants are removed every year [8]. Peri-implantitis is the major cause of the implant retrieval and also the most common complication caused by implant surgery. Mombelli et al. reported that plaque formation can occur on dental implant surfaces similar to that of tooth surfaces [9].

The mainstay treatment for peri-implantitis includes the elimination of etiologic factors and the mechanical removal of calculus, cement, and plaque followed by subgingival irrigation with tetracycline and chlorhexidine base mouthwash. Lang et al. first described a treatment protocol for peri-implantitis including mechanical cleaning, decontamination of the implant surface, antibiotic regimen, and regenerative surgery (if required) in 1997 [10]. However, literate indicates that the use of air-powder abrasive (APA) treatment for the decontamination of the implant surface remains controversial. Although some reports advocate that the in vivo usage of APA systems pose a potential risk of emphysema and may have limited clinical applications [11, 12], some other studies, such as the study reported by Duarte et al. found that APA is more effective in the decontamination of dental implants than lasers, metal curettes, and plastic curettes [13]. On the other hand, Renvert et al. compared the use of APA and Er:YAG laser application on dental implant surfaces and found that the two methods produced similar outcomes with regards to the decontamination of implant surfaces. In the present study, we used a combination of APA and citric acid for the decontamination of the surfaces of the retrieved implants, mainly because both methods are easily available, have minimal cost, and are easy to use when compared to laser treatment [14].

Another controversy reported in the literature is concerned with the re-healing process around the contaminated implant surface. Although some studies contend that re-healing is possible around the dental implants affected by peri-implantitis depending on the implant surface treatment modalities employed prior to re-insertion [15, 16], some other studies, such as the study reported by Persson et al. showed that they did not detect any re-osseointegration around the contaminated non-modified surface of the dental implants after the treatment of the implant surfaces affected by peri-implantitis [17]. On the other hand, Hürzeler et al. detected re-osseointegration with guided bone regeneration [18], Persson et al. found re-osseointegration in 84% of SLA implants [19], and Alhag et al. showed re-osseointegration on plaque-covered implant surfaces after the removal of the plaque by means of citric acid, tooth brush, and hydrogen peroxide [20].

Levin et al. conducted a similar study and investigated the success rate of retrieved dental implants that were re-implanted into dogs. The infected implants were re-implanted into dog jaws without any chemical or mechanical cleaning and the authors reported that there was no difference in terms of BIC percentage between the infected/reinserted and new dental implants after an appropriate healing period [21]. In our study, the experimental groups were formed in line with the literature; in group 1, a highly effective decontamination method (APA and citric acid) was used [22], whereas in groups 2 and 3, autoclave sterilization was used for the decontamination of implant surfaces since autoclave is the most common method for the sterilization of surgical instruments and is widely used in dental implant laboratory studies [23]. After the experiment, it was revealed that autoclave sterilization does not interfere with osseointegration, which implicates that autoclave sterilization is a useful method to be used in re-implantation procedures.

The main purpose of the current study was to evaluate the degree of osseointegration in the dental implants inserted for a second time following the treatment of the implant surfaces with peri-implantitis therapy. Unlike other peri-implantitis therapy approaches, the approach developed in this experiment allowed all the treatment procedures to be performed outside the mouth, thereby enabling the decontamination methods to be applied easily and uniformly to all the regions of the implants in a more standardized manner than would be possible intra-orally.

## Conclusions

In conclusion, the results of this study indicated that there was no significant difference in the BIC percentages and the RFA measures between the implants retrieved due to peri-implantitis and re-implanted in the dog jaws and the new dental implants inserted for the first time. Moreover, the results also suggested that a dental implant retrieved due to peri-implantitis may be re-used in the same patient after decontamination of the implant surface. Nevertheless, despite the encouraging findings presented by this study, further studies including a larger number of implants are needed to substantiate the findings of our study.

## Abbreviations

APA: Air powder abrasive
ARRIVE: Animal Research Reporting in Vivo Experiment
BIC: Bone-implant contact
IM: Intramuscular
IV: Intravenous
RFA: Resonance frequency analysis
SLA: Sandblasted and acid-etched
